# A Rare Case of Aortic Valve Thrombosis 12 Years Post-Bioprosthetic Implantation

**DOI:** 10.1016/j.jaccas.2025.103934

**Published:** 2025-07-09

**Authors:** Zeyad Kholeif, Rana Rashwan, Omar Aboukhatwa, Tim Leiner, Sorin Pislaru, Bas Kietselaer

**Affiliations:** a Department of Cardiology, Mayo Clinic, Rochester, Minnesota, USA; b Department of Radiology, Mayo Clinic, Rochester, Minnesota, USA

**Keywords:** aortic valve, computed tomography, echocardiography

## Abstract

**Background:**

Bioprosthetic valve thrombosis (BPVT) can occur after valve implantation, typically within the first 3 months. However, it can also develop after many years, as shown in this case.

**Case Summary:**

We report a 75-year-old male who developed progressive heart failure and cardiogenic shock 12 years after receiving a bioprosthetic aortic valve. Echocardiography revealed a thickened, immobile echo density on the valve, suspicious for BPVT, which was confirmed by cardiac computed tomography. The patient was treated with oral anticoagulants which successfully reduced the valve gradient.

**Discussion:**

This case shows that, although uncommon, BPVT can occur late after valve implantation. Additional risk factors such as atrial fibrillation without adequate anticoagulation can contribute to late BPVT. Anticoagulation is the mainstay of treatment. This case highlights the importance of considering BPVT in long-term bioprosthetic valves because it has important therapeutic and prognostic consequences.

**Take-Home Message:**

Bioprosthetic valve thrombosis 12 years after implantation is possible, especially in patients in a pro-coagulant state.

## Visual Summary

**Figure:**
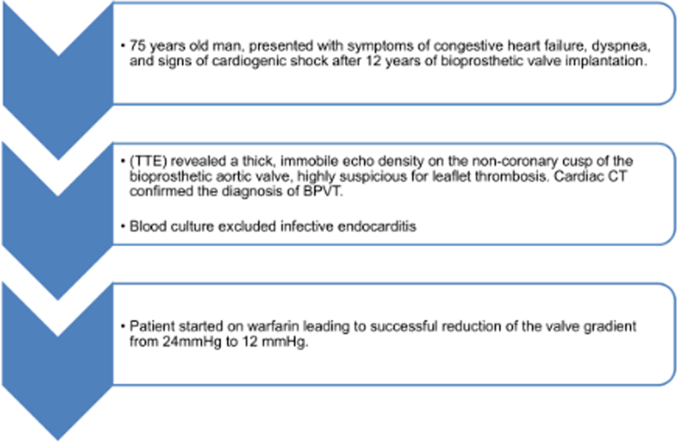

## Clinical presentation

A 75-year-old male with a history of bioprosthetic aortic valve replacement presented to the emergency department with worsening shortness of breath, progressive congestive heart failure, and signs of cardiogenic shock. On examination, his temperature was normal, heart rate was 158 beats/min, respiratory rate was 27 breaths/min, and oxygen saturation was 88%. The patient was placed on 2 L of oxygen via nasal cannula. The electrocardiogram showed new-onset atrial flutter with rapid ventricular response. The patient started intravenous fluids and 20 mg of intravenous diltiazem (as he was already on oral diltiazem at home). Given the concerns for acute decompensated heart failure and elevated lactate, he was transferred to the cardiac intensive care unit for further management.

**Take-Home Messages**

• Bioprosthetic valve thrombosis after a decade is a serious complication.
• Multimodality imaging is crucial to look for features such as thickened valve leaflets and decreased leaflet mobility and is essential in diagnosing patients with bioprosthetic valve thrombosis.
• Initial treatment with anticoagulation can help with both diagnosis and subsequent management.

## Past medical history

In 2012, the patient underwent an aortic valve replacement with a 23-mm Carpentier-Edwards Perimount bioprosthetic valve. He also has chronic hypertension, hyperlipidemia, and a single episode of self-limiting postoperative atrial fibrillation in 2012, not on anticoagulation.

## Investigations

**Figure 1:**
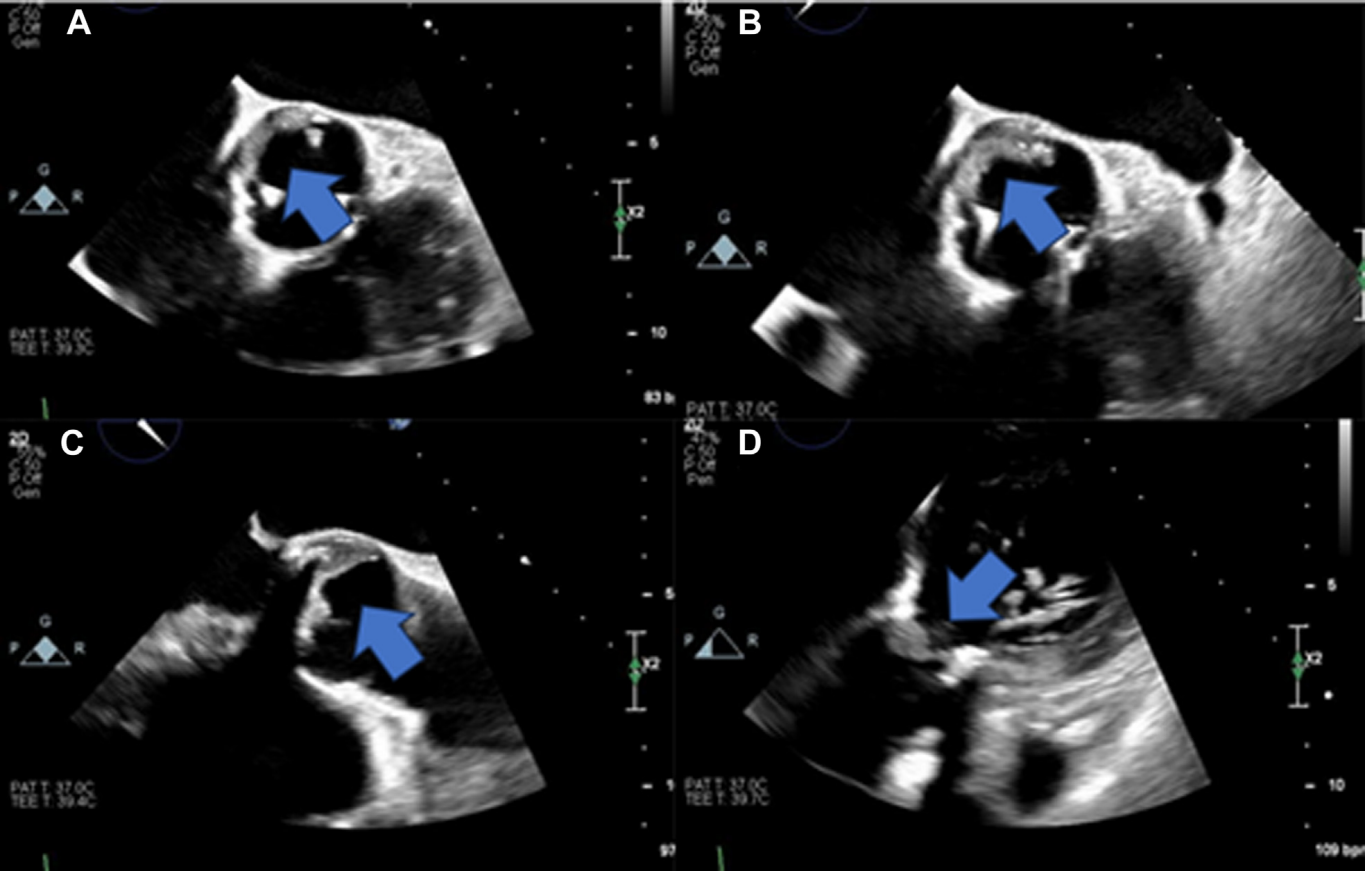
Transesophageal Echocardiogram A thick immobile echo density is shown along the noncoronary positioned bioprosthetic aortic valve cusp with restricted leaflet motion which suggests leaflet thrombosis.

**Figure 2:**
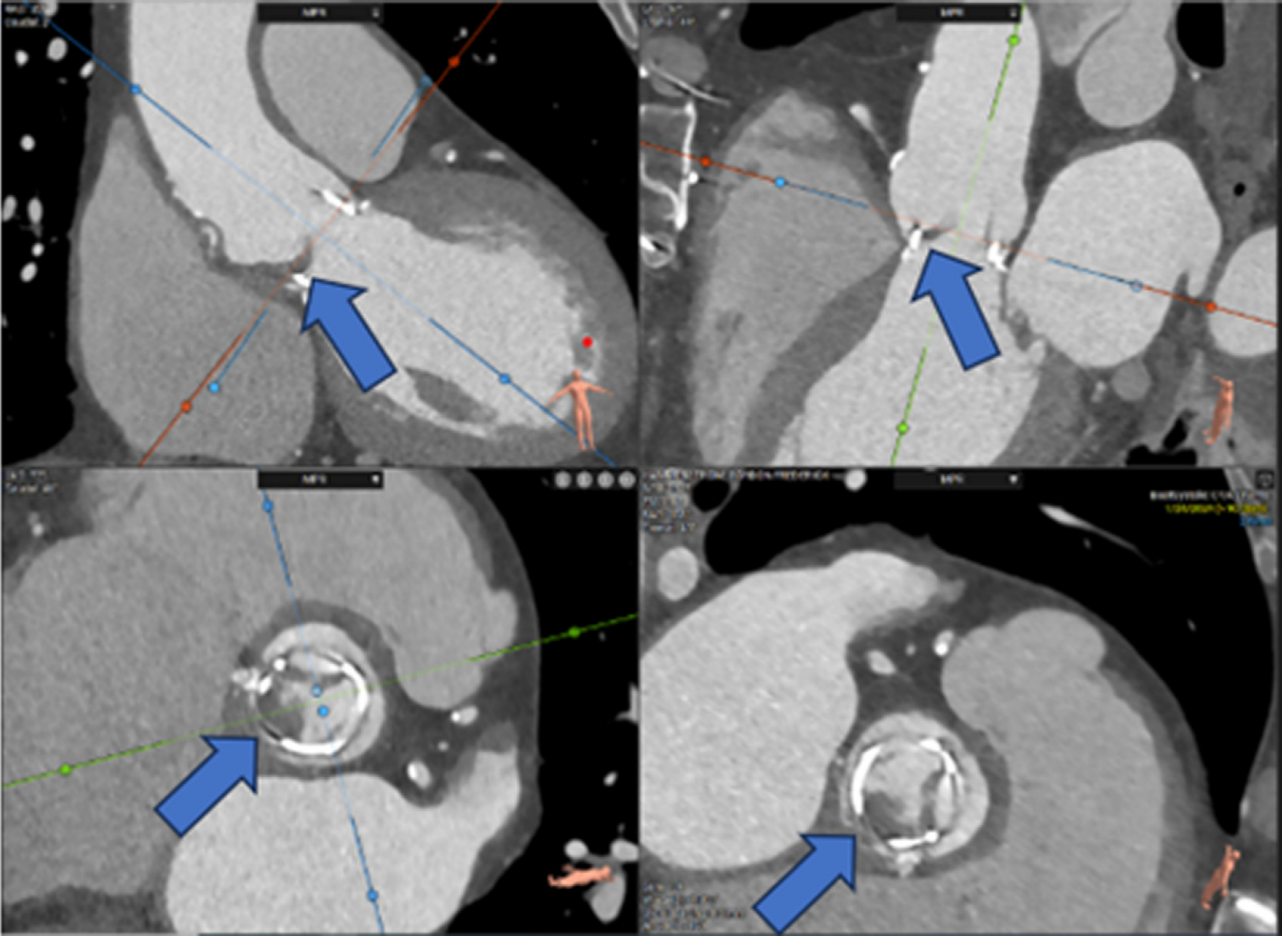
Cardiac Computed Tomography A noncalcified bulky thrombus of the bioprosthetic aortic valve is shown that is greater on the arterial side of the noncoronary cusp with subtotal occlusion of the noncoronary sinus of Valsalva.

Transthoracic echocardiography (TTE) revealed a newly reduced left ventricular ejection fraction of 30% and an increased prosthetic valve mean gradient of 17 mm Hg. A transesophageal echocardiogram (TEE) revealed a thick, immobile echo density on the noncoronary cusp of the bioprosthetic aortic valve, highly suspicious for leaflet thrombosis (Figure 1, Video 1). Cardiac computed tomography (CT) confirmed the diagnosis of bioprosthetic valve thrombosis (BPVT) and revealed a subsegmental pulmonary embolism (Figure 2).

**Visual Summary:**
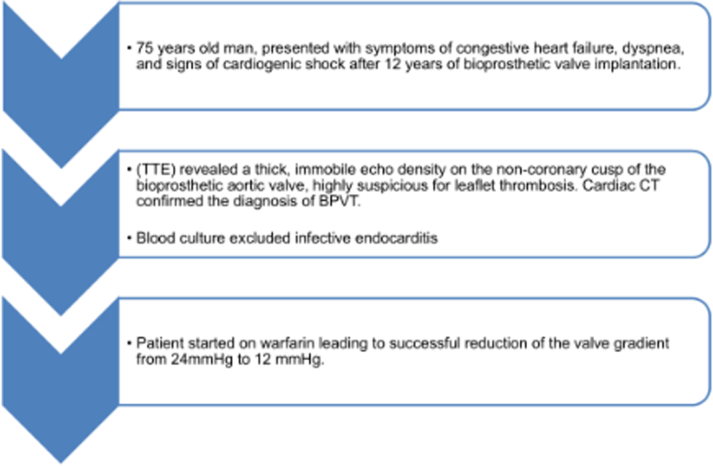
Visual Summary of Late-Onset BPVT

## Differential diagnosis

Blood cultures were initially taken to rule out infective endocarditis, with negative results. Malignancy was suspected due to various thrombotic events but was excluded based on chest and abdomen imaging. A hematology workup, including an antiphospholipid study, returned negative results. A peripheral smear was performed to evaluate thrombocytopenia, and the result did not show any concern for schistocytes or platelet clumping.

## Management

Given the findings, heparin therapy was initiated, and anticoagulation was escalated to warfarin with a target INR of 2.0 to 3.0 for stroke prevention. The patient was managed with intermittent intravenous diuresis and guideline-directed medical therapy. During hospitalization, complications included transient ischemic attacks, anemia, and abdominal hematoma, all managed appropriately.

## Outcome and follow-up

One week later, a TTE showed a reduction in valve gradient from 24 mm Hg to 12 mm Hg after initiation of anticoagulation, confirming success of anticoagulant treatment. The patient was discharged in stable condition after 2 weeks and recovered well.

## Discussion

Bioprosthetic valve replacement is a preferred option for patients because it avoids long-term anticoagulation and has a lower incidence of BPVT. However, studies have shown that BPVT occurs more frequently than previously thought. Oliver et al^1^ identified BPVT in 10 of 161 (6.2%) patients with bioprosthetic valve dysfunction, and Egbe et al^2^ found BPVT in 46 of 397 (11%) bioprosthetic valves explanted at Mayo Clinic, where 30 of 46 (65%) cases occurred >12 months post-implantation, highlighting a higher-than-expected incidence of BPVT. BPVT is typically considered a complication that occurs within the first 3 months of implantation,^3^ but a recent case series has shown a peak incidence of BPVT between 13 and 24 months, challenging the earlier belief that BPVT is confined to the first 3 months post-valve implantation.^2^, ^3^, ^4^, ^5^ This case is particularly rare, as BPVT developed 12 years after implantation, making it an exceptionally unusual occurrence in the context of bioprosthetic valve dysfunction.

Clinical presentations of BPVT can vary from an incidental finding in an asymptomatic patient to severe symptoms including dyspnea, syncope, acute heart failure, or cardiogenic shock.^5^^,^^6^ Clinical features supportive of BPVT include new-onset heart failure, a rapid rise in the mean prosthetic gradient compared with an initial normal postoperative study, presence of risk factors for BPVT, and new systemic thromboembolic events.^6^ In this case, the patient presented with worsening shortness of breath, congestive heart failure, and signs of cardiogenic shock, all of which were consistent with valve dysfunction due to BPVT.

Risk factors for BPVT include atrial fibrillation without anticoagulation therapy, low cardiac output state, and a history of hypercoagulable disorder.^2^^,^^6^ In this case, malignancy was initially suspected due to thrombotic events but was ruled out through imaging and hematology workup was unremarkable. The patient’s identified risk factors were atrial fibrillation without adequate anticoagulation therapy and low output state with a left ventricular ejection fraction of 30%. Potential late-onset risk factors include valve degeneration (calcification), hyperlipidemia, and poor left ventricular function; however, more research is needed to confirm these risk factors.

Diagnosis relies on multimodal imaging, which can include both TEE/TTE and cardiac CT. Distinguishing BPVT from other causes of bioprosthetic valve failure, such as structural valve degeneration or endocarditis, is essential for proper management. BPVT is suggested when an acute rise in transvalvular gradient of more than 50% above the baseline is observed within 5 years, valve leaflet thickening more than 2 mm (especially in the downstream aspect of the bioprosthetic valve), and/or when there is restricted cusp mobility noted on echocardiography. If all criteria are met, BPVT diagnosis has a sensitivity of 76% and specificity of 93%.^2^ The regression of echocardiographic abnormalities after anticoagulation treatment further supports the diagnosis of BPVT.^6^ In this case, the patient’s mean prosthetic gradient decreased from 24 to 12 mm Hg after anticoagulation, confirming the BPVT diagnosis. CT has also been used to detect BPVT. Reduced leaflet motion and hypoattenuated leaflet thickening are characteristic of BPVT.^7^ In this patient, both TEE and cardiac CT were used. TEE in patients with prosthetic valves may be complicated by acoustic shadowing caused by the prosthesis. Therefore, in addition to TEE and blood cultures, multimodality imaging in establishing a diagnosis in patients with prosthetic valve failure is important and recommended by current guidelines.^8^

Treatment of BPVT includes oral anticoagulants including heparin or vitamin K antagonists. Early initiation of oral anticoagulants is crucial to prevent thromboembolism and valve degeneration. There are limited data on novel oral anticoagulants in the treatment of BPVT. One study, however, has shown promising results with the use of novel oral anticoagulants. In this study, 106 patients with subclinical BPVT were treated with novel oral anticoagulants — it was noted that novel oral anticoagulants were as effective as vitamin K antagonists in the treatment of BPVT.^9^ In cases of BPVT while on therapeutic vitamin K antagonists with an INR between 2 and 3, aiming for a higher INR target of 2.5 to 3.5 can be considered.^10^

## Conclusions

This case underscores the importance of considering valve thrombosis in patients with bioprosthetic valves, even years after implantation. Prompt echocardiographic assessment is recommended for patients presenting with shortness of breath, heart failure, and a history of bioprosthetic valve implantation to look for BPVT. Cardiac CT can serve as a complementary imaging modality to confirm the presence of BPVT. Once diagnosed, early initiation of oral anticoagulants with close follow-up and echocardiographic assessment is essential to assess the response to treatment.

## Funding Support and Author Disclosures

The authors have reported that they have no relationships relevant to the contents of this paper to disclose.

## Appendix

Video 1 2-Dimensional Views (A) Two-dimensional short-axis view of the aortic valve showing thick immobile echo density noted along the noncoronary positioned bioprosthetic aortic valve cusp with restricted leaflet motion, suggestive for leaflet thrombosis. (B) Two-dimensional long-axis view of the aortic valve showing thick immobile echo density noted along the noncoronary positioned bioprosthetic aortic valve cusp with restricted leaflet motion which suggests leaflet thrombosis.

## References

[1] Oliver J.M., Galloge P., Gonzalez A., Dominguez F.J., Gamallo C., Mesa J.M. (1996). Bioprosthetic mitral valve thrombosis: clinical profile, transesophageal echocardiographic features, and follow-up after anticoagulant therapy. J Am Soc Echocardiogr.

[2] Egbe A.C., Pislaru S.V., Pellikka P.A. (2015). Bioprosthetic valve thrombosis versus structural failure: clinical and echocardiographic predictors. J Am Coll Cardiol.

[3] Heras M., Chesebro J.H., Fuster V. (1995). High risk of thromboemboli early after bioprosthetic cardiac valve replacement. J Am Coll Cardiol.

[4] Pislaru S.V., Hussain I., Pellikka P.A. (2015). Misconceptions, diagnostic challenges and treatment opportunities in bioprosthetic valve thrombosis: lessons from a case series. Eur J Cardiothorac Surg.

[5] Egbe A., Pislaru S.V., Ali M.A. (2018). Early prosthetic valve dysfunction due to bioprosthetic valve thrombosis: the role of echocardiography. JACC Cardiovasc Imaging.

[6] Puri R., Auffret V., Rodés-Cabau J. (2017). Biprosthetic valve thrombosis. J Am Coll Cardiol.

[7] Hansson N.C., Grove E.L., Andersen H.R. (2016). Transcatheter aortic valve thrombosis: incidence, predisposing factors, and clinical implications. J Am Coll Cardiol.

[8] Zoghbi W.A., Jone P.N., Chamsi-Pasha M.A. (2024). Guidelines for the evaluation of prosthetic valve function with cardiovascular imaging: a report from the American Society of Echocardiography developed in collaboration with the Society for Cardiovascular Magnetic Resonance and the Society of Cardiovascular Computed Tomography. J Am Soc Echocardiogr.

[9] Chakravarty T., Søndergaard L., Friedman J. (2017). Subclinical leaflet thrombosis in surgical and transcatheter bioprosthetic aortic valves: an observational study. Lancet.

[10] (2015. Accessed February 12, 2025). Prosthetic valve thrombosis: Time is critical. MayoClinic.org.

